# Supplementary figures and images for: The human dorsal anterior cingulate facilitates acceptance of unfair offers and regulates inequity aversion
Source: PLoS Biol. 2026 Feb 5;24(2):e3003007. doi: 10.1371/journal.pbio.3003007 (PMC12875481; doi:10.1371/journal.pbio.3003007)

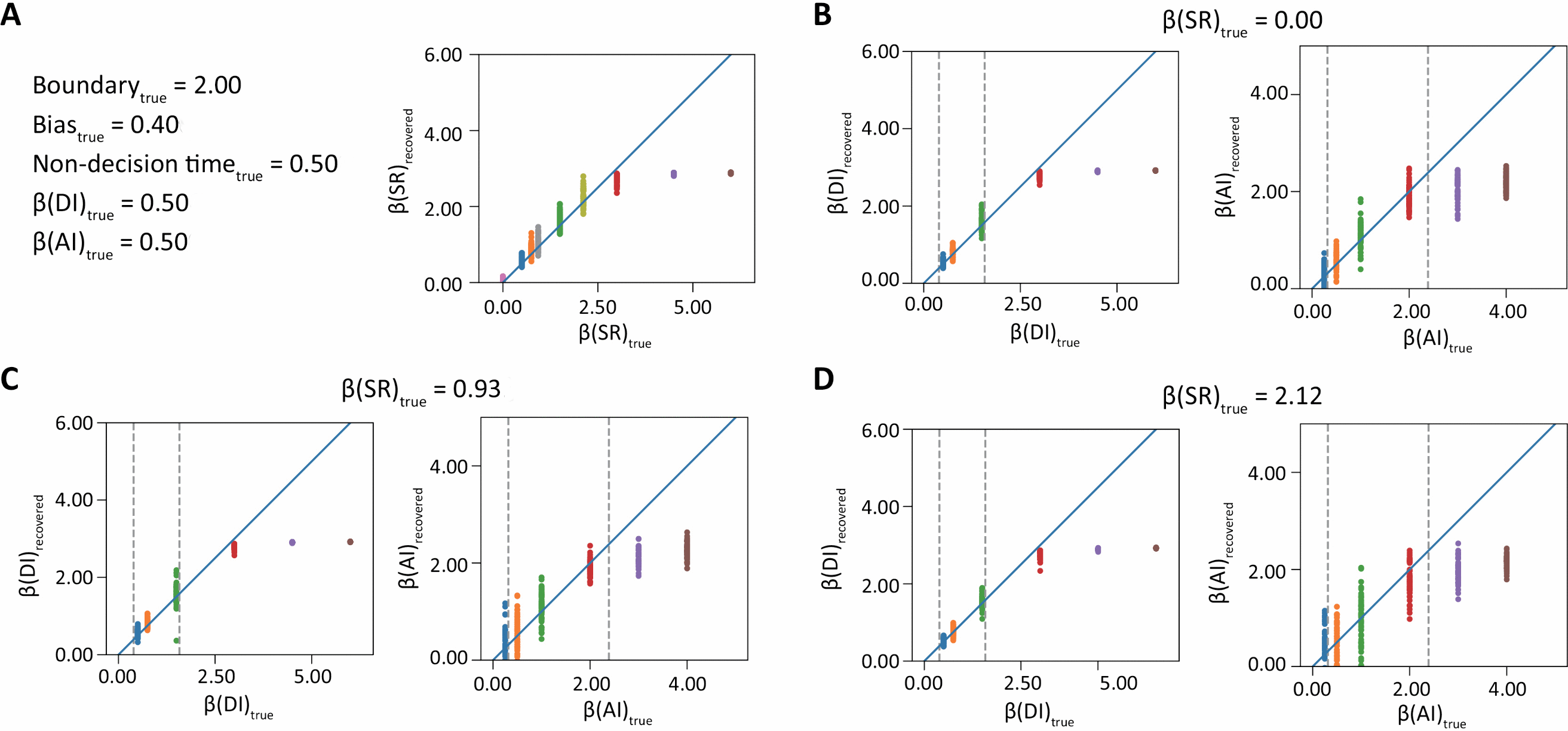

Supplement: S2 Fig — We examined the parameter ranges that can be accurately estimated by our model. (A) Simulated reward-seeking agents with variable β(SR). We evaluated the recovery of β(SR) while keeping the boundary, bias, nondecision time, β(DI), and β(AI) at the displayed typical values. The recovered β(SR) plateaued when its true value exceeded 3.00. Therefore, this does not pose a practical problem, as the maximum β(SR) observed across participants was 2.12. Other parameters were estimated accurately, as shown in Fig 2F. We then simulated inequity-aversive agents with variable β(SR), β(DI), and β(AI) through B to D. We assessed the recovery of β(DI) and β(AI), while varying β(SR) within a reasonable range (0.00 in B, 0.93 in C and 2.12 in D). The recovered β(DI) and β(AI) also plateaued when their true values were larger than 3.00. Again, this does not present a practical issue, as the maximum β(DI) and β(AI) observed across participants were 1.58 and 2.39, respectively. Other parameters were estimated accurately, as in Fig 2F. (TIF) [file pbio.3003007.s002.tif]
