# Supplementary material for: The human dorsal anterior cingulate facilitates acceptance of unfair offers and regulates inequity aversion
Source: PLoS Biol. 2026 Feb 5;24(2):e3003007. doi: 10.1371/journal.pbio.3003007 (PMC12875481; doi:10.1371/journal.pbio.3003007)
Supplement: Attachment — Submitted filename: PLOSBioResponseToReviewer.docx [file pbio.3003007.s033.docx]

**Response to Reviewer #1**

**Comment:** This is an innovative study of an understudied but important area of cognitive neuroscience, namely how to understand the motivations underpinning (altruistic) punishment and their neuroanatomy.

***Response:***

We are grateful to the reviewer for the positive evaluation of our paper.

**Comment:** My main concerns are the following:

1) The interpretation of results as reflecting suppression of emotions is not being directly tested by the experiment and therefore a descriptive rather than mechanistic conceptualisation should be used with mechanistic interpretations discussed as potential explanations for the findings:

1a) Abstract, Intro and Discussion: "suppressing" emotions is not really measured as it could also be that the anger driving inequity aversion is replaced by another emotion, e.g. the anticipated monetary reward value of accepting the offer. I would therefore use a more descriptive term instead of "suppression", such as "overcoming" or "replacing" or "deciding against".

***Response:***

We agree with the reviewer that we did not directly prove “suppression”. Therefore, we changed the title of the paper to “Dorsal anterior cingulate regulates inequity aversion and facilities acceptance of unfair offers” (page 1, line 1). In the Abstract, we used the expression “internal mental effort when deciding to accept unfair offers,” as suggested (page 1, line 18).

**Comment:** 1b) at the end of the introduction, long response times are equated with suppressing negative feelings, but an equally likely and more plausible explanation in my view would be higher levels of choice ambivalence which would also align well with the dACC activations when conflicting choices are introduced (Botvinick et al), as well its more general role in salient feelings with behavioural relevance such as social and physical pain (Eisenberger's work).

***Response:***

Following the comment, we have revised the last part of the Introduction as follows (page 4, line 14).

"Based on previous studies on cognitive control (i.e., conflict detection and resolution) [34–40] and salient feelings with behavioral relevance such as social and physical pain [41,42], we further hypothesized that the anterior cingulate cortex plays a crucial role in this process."

Although we excluded the possibility of simple conflict monitoring by including response times as a regressor, we described broader potential contributions of the dACC in the revised manuscript.

**Comment:** 2)The computational analysis methods are sophisticated and I am not an expert on computational modelling. Nevertheless I would expect the authors to explain their methods in a way which can also be understood by someone who is only familiar with standard statistical approaches. From a standard statistical perspective, there were several concerns:

2a) It appears that the authors first optimized their models as presented in the model selection results and then used the best model for their further analyses, I am only familiar with prediction modelling where this would be seen as inflating the significance of the final analysis (double-dipping), could the authors address this please?

***Response:***

To avoid data overfitting (double-dipping), we conducted a model selection that theoretically aims to optimize predictive performance using the widely applicable information criteria (WAIC). To validate this method, we confirmed the generalizability by the (1) cross validation of response times and (2) parameter recovery test as follows.

(1) Cross validation of response times

We evaluated generalization (prediction) performances. Regardless of the reward ratio in each trial, the predicted response times closely matched the observed values, and there were no indications of overfitting (**Figure 2C**).

(2) Parameter recovery test

We prepared two synthetic (participant) parameter sets representing (i) a “reward-seeker” and (ii) an “inequity-avoider” and generated choices and response times from each set. We then applied our model selection method and examined whether the method could recover the parameter sets without overfitting. As a result, the method successfully recovered the true parameter values accurately for both sets (**Figure 2F and 2G**). These results confirmed that our model selection procedure yields unbiased parameter estimates and does not spuriously inflate the significance.

Accordingly, we expanded the Materials and Methods section (**Model validation of the best model**) (page 32, line 11) to describe these procedures and updated the Results section (page 10, lines 11 and 19).

**Comment:** 2b) There were multiple statistical results presented, but no multiple comparison correction.

***Response:***

We re-conducted all relevant statistical tests with the Holm–Bonferroni correction. Adjusted p-values are reported in the Results section.

**Comment:** 3) Terminology - and connection with the wider literature: "inequity aversion" should be linked with the existing literature on altruistic punishment and indignation / moral anger

***Response:***

In the revised Introduction section (page 2, line 18), we described the connections between inequity aversion, altruistic punishment, and moral outrage to broaden the context of inequity aversion. As a result, the following three references are cited in the revised manuscript.

- Darley, JM and Pittman TS. (2003) The Psychology of Compensatory and Retributive Justice.
- Quervain, et. al, (2004) The Neural Basis of Altruistic Punishment.
- Fehr, E and Gachter, S (2002) Altruistic punishment in humans.

**Comment:** 4) As standard fMRI sequences lead to large signal loss in brain regions relevant for emotions such as the orbitofrontal cortex, it will be important to discuss this in the conclusions and add images of the implicit mask produced by SPM to the supplement to show the coverage of orbitofrontal areas and describe the areas not covered.

***Response:***

To visualize the scan coverage, we generated subject-specific implicit masks and averaged them to produce a group-level coverage map (**S8 Figure**; see also Methods, page 33, line 14). We displayed this map alongside the standard anatomical template. We found that the coverage of the amygdala and surrounding medial temporal areas are largely intact, while the signal in the orbitofrontal cortex (OFC) was partly lost due to susceptibility artifacts. We described this as a limitation in the Discussion section (page 25, line 5)

**Comment:** Minor comments:

5) abstract: was it bilateral vlPFC?

***Response:***

We thank the reviewer for the comment. We clarified that our PPI analysis revealed significant connectivity between the dACC and bilateral vlPFC in the Abstract (page 1, line 25).

**Response to Reviewer #2:**

**Comment:** Overall evaluation: While there are elements to like about this study, including a relatively large sample size, a computational approach, and an interesting question, I found my enthusiasm tempered by several factors. Perhaps largest among these is the overinterpretation of results, largely based on reverse inference, showing that the ACC is necessary to overcome pride, or otherwise is performing an emotion regulation role. I also felt that at a number of points there is a lack of clarity about key methods that made it hard to know exactly what the authors did, and what they found. I also think the authors need to do much more to exclude alternative explanations for their results. Finally, while I think the computational modeling approach is admirable, I think much more could and should be done to unpack these results and to understand what they mean. What follow are my more specific points on each of these issues.

***Response:***

We thank the reviewer for pointing out problems in our original submission; namely, the overinterpretation of the results and the lack of methodological details. Accordingly, we have conducted additional analysis and thoroughly revised the manuscript as explained in our replies to the comments below.

**Comment:** 1.	My biggest concern in the paper is what feels like an attempt to "oversell" the results. The title of the paper claims to show that the ACC is necessary to overcome pride in the Ultimatum Game. The introduction and discussion similarly suggest that what the authors have shown is that the interactions between ACC, vlPFC, and amygdala are necessary for "suppressing the negative response to inequity in the amygdala" and that these results may contribute to understanding well-being. I don't think such claims are well-justified given the design of the study. At no point is pride, or any other emotion, measured. Nor is there really any way to know what role the ACC is performing in this study (see my comments below). Instead, the paper engages in quite a lot of reverse inference to arrive at a connection between pride, negative emotion, and emotion regulation. Given that the ACC region the researchers report is frequently observed for all sorts of conflict-related tasks and its function is hotly debated, I didn't feel confident that the authors' interpretation was justifiable. They'd likely need to tone down their interpretation considerably, and stick much closer to the data.

***Response:***

We are grateful to the reviewer for the constructive criticisms and helpful suggestions. Our data showed that participants who accepted unfair offers (i.e., not inequity averse in terms of choice) with long response times exhibited heightened dACC activity, which had negative connectivity with the vlPFC, whose own activity was synchronized with activity in the amygdala when disadvantageous inequity was large. In addition, functional connectivity between the dACC and the vlPFC explained both choices and response times for disadvantageous offers. Therefore, we changed the title of the paper to “Dorsal anterior cingulate regulates inequity aversion and facilities acceptance of unfair offers” and removed not only the term “pride” but also “emotion” from the title and abstract. Furthermore, as detailed below, we made our best effort to avoid reverse inferences in the revised manuscript.

**Comment:** 2.	My second, related issue concerns a set of alternative interpretations of the results that are perhaps much less 'interesting' but more plausible, in my view. First, research suggests that areas related to conflict and cognitive control, including the ACC, may respond more strongly when there is greater conflict, and when RTs are longer, and may even track the degree of evidence accumulation being performed during social decisions (Hutcherson & Tusche, 2022). The authors do not control for RTs in any of their analyses, but it is clear from their modeling that DI is likely strongly correlated with response time in this study. This is less likely to be true (or at least, could be less true) in people who are less bothered by DI (i.e., have a lower weight on this factor). Thus, one could observe a modulation of the association between DI and ACC response as a function of the weight on DI not because the ACC somehow causally reduces pride, causally regulates negative emotion, or even causally changes rejection rates, but simply because other factors shaping these things could increase (or decrease) the relative conflict (or RT) associated with high and low-DI trials. This could similarly explain the connectivity results. Without reporting a much more extensive assessment of how DI (or SR) correlates with trial-by-trial response times and response conflict (either in observed behavior, or in simulations from DDMs), and showing that this does not account for the pattern of results, I have difficulty trusting the authors' preferred interpretation. At minimum, I would want to know whether these results survive after controlling for RTs. I'd also want to know whether the ACC region the authors are finding shows evidence that it is simply tracking RTs or simply correlating with overall evidence accumulation signals.

***Response:***

We are grateful to the reviewer for pointing out this important issue. In accordance with the reviewer’s suggestion, in our revised analysis, we included trial-by-trial response times in addition to DI as a parametric modulator and found a more robust association between DI and the ACC response weighted by the DI coefficient compared with the original analysis (**Figure 4A** and page 14, line 18). We think that this result rules out the possibility that relative conflict or RT is the cause of the ACC response we report.

Additionally, a second‐level contrast of the RT regressor alone yielded no significant clusters in the ACC or elsewhere. These results indicate that variation in response speed does not drive our effects. Notably, the ACC cluster associated with overcoming inequity was even more pronounced in this response-time‐controlled model, further strengthening our original interpretation. These findings confirm that dACC activation reflects the process of “overcoming inequity” per se, rather than simply longer decision times or generic conflict‐related evidence accumulation. We have updated the results and **Figure 4A** to illustrate these controls.

**Comment:** 3a.	The authors could also provide much more detail on the DDM results themselves. First, 500 burn in and 500 samples seems like a relatively small amount of fitting for an hSSM model. It is comforting to see the model produce aggregate level results that capture basic patterns, but that is a far cry from saying that the model fits well and converges to a point where we can have confidence in the estimated values of individual-level parameters. Can the authors report parameter recovery statistics, and convergence statistics of the winning model (e.g., Gelman-Rubin diagnostics for all parameters).

***Response:***

In response to the comment, in the revised analysis, we discarded the first 15,000 iterations as burn-in and retain 5,000 post–burn-in samples for each chain (previously 500 burn-in/500 samples). Rerunning the HSSM with these settings yielded both group-level and individual-level parameter estimates, which is identical to the original results, demonstrating the robustness of our findings. We additionally computed Gelman–Rubin statistics for all chains and found that all statistics were below 1.1, indicating the satisfactory chain convergence. These changes in the analysis are explained in the Materials and Methods section (page 30, line 8).

We also reported the parameter recovery statistics (**Figure 2E and 2F**, and page 11, line 11).

**Comment:** 3b.	Second, the authors never provide any details about what the values of the best-fitting parameters are. This would provide a lot of interesting information for the reader, such as how self-interest vs. DI vs. AI are weighted, whether there is a starting bias to reject (or accept) offers overall, and whether things like the threshold might also be correlated with RTs (it should be). I'd be particularly interested to know whether and how thresholds and starting biases correlate with any of the observed effects.

***Response:***

We added the details of β(DI), β(AI),β(SR), bias, and boundary in **Figure 3** and supporting information (**S7 Figure**), as suggested. The results are also explained in the main text (page 13, line 12, page 14, line 1) and the Materials and Methods section (page 32, line 4). These results replicated patterns we already reported in our previous study (Numano & Haruno, 2025).

**Comment:** 4.	The authors are similarly elliptical about the fMRI analyses. Although I think I understood the basics of their primary GLM, I was left with a number of questions about the PPIs that they report. They mention performing a gPPI, which I take to mean that they included all original regressors from the primary GLM, and then examined the neural activity associated with the PPI as well, but it is not 100% from their methods whether this is the case. Similarly, it was unclear to me whether ALL PPIs (including the ACC and vlPFC PPIs) were essentially interacting the neural signatures with the parametric regressor for DI. For instance, for the vlPFC connectivity, sometimes they simply say that they looked at connectivity "at offer time", and at other times it sounds as if they are looking at connectivity modulated by DI. In general, being clearer about what was done would be helpful.

***Response:***

We apologize for the misleading descriptions of the generalized PPI analyses in our original manuscript. As the reviewer correctly assumed, for each seed region (the dACC and the vlPFC), we included all individual‐level regressors from our primary GLM (GLM1), in addition to the physiological time series from the seed. In all PPI analyses, the psychological regressor was the DI parametric modulator (i.e., trial‐by‐trial DI values), which was multiplied by the seed’s time series to form the PPI regressor. Thus, we specifically tested how connectivity between the seed and target regions was modulated by the magnitude of disadvantageous inequity at the offer onset. This procedure was applied in both the ACC-vlPFC and ACC-amygdala connectivity analyses. In other words, we consistently examined DI-modulated coupling at the timing of the offer throughout our gPPI analysis. We explicitly explained these points in the Materials and Methods section of the revised manuscript (page 36, line 4, line 11, line 18).

**Comment:** 5.	The authors argue that accepting unfair offers takes more "regulation" than rejecting them, but all of the analyses they report where neural activity or connectivity is correlated with RTs involves simply the raw response time, rather than the response time difference to accept vs. reject. There's a lot that can go into a raw response time, including a participant's simple motor speed, the threshold they've got for a response, they're starting bias, etc. None of these have to do with overcoming negative emotion or pride. So I'm not clear on why the raw response time is the appropriate measure here, rather than the difference between acceptance and rejection. Do the results hold if that difference is used instead of the raw RT?

***Response:***

We agree with the reviewer that raw response times reflect too many different factors. Since calculating the difference between accepting and rejecting RT was not feasible, as some participants did not produce any rejection in this task, we adopted an alternative normalization approach. For each participant, we computed the mean RT on neutral (50:50) offers and subtracted this value from their accepting and rejecting RTs in unfair trials. This adjustment likely controls individual differences in the baseline motor speed and response threshold (page 37, line 8).

Using these normalized RTs, the correlation with the connectivity between the dACC and vlPFC remained significant (**Figure 4E**). In contrast, the previously reported correlation between response time and the vlPFC-amygdala connectivity did not survive with this normalization. We have therefore removed the scatter‐plot panels in **Figure 5**, retaining only the dACC-vlPFC connectivity plot. We updated the Results (page 18, line 4, page 19, line 16) and the Materials and Methods sections (page 37, line 8) accordingly. These changes have ruled out the possibility that our results arose from general response-speed differences in participants.

**Comment:** 6.	The authors never report basic neural correlates of parametric modulators for SR, DI, or AI, so it is not clear whether the observation of ACC modulation and connectivity, or of vlPFC to amygdala connectivity modulated by DI has any real relevance to DI processing. This is especially problematic for the author's conceptual diagram, which suggests that ACC modulates vlPFC which modulates amygdala and then alters choice. If the amgydala isn't correlating with DI to begin with, or if its response to DI is not correlated with the weight on DI, then simply observing connectivity to vlPFC (which itself isn't obviously correlated with DI, or with accepting or rejecting offers) doesn't tell you that the vlPFC is somehow regulating negative emotion, directed by the ACC. Amygdala connectivity with the vlPFC is also only identified using small-volume correction with a fairly liberal threshold, and does not correlate with rejection rates (though does correlate with raw response times) so the result feels a little bit like it was manufactured so as to draw a connection to emotional processing, rather than like it reflects robust evidence for the authors' model.

***Response:***

To address this comment, we contrasted each parametric modulator (SR, DI, AI, and trial‐by‐trial RT), but none of these contrasts yielded significant clusters at cluster-level FWE corrected p < 0.05. This observation suggested that no variable robustly drives whole‐brain activation under strict correction.

We then aimed to decide whether the amygdala tracks DI. We split participants at the median of their individual β(DI) estimates and examined neural activity correlated with DI in the higher β(DI) group. This analysis revealed a significant cluster in the amygdala (**S4 Figure** and **S4 Table**; we conducted small volume correction for multiple comparisons (cluster-level FWE corrected p < 0.05) within a bilateral amygdala ROI). This result demonstrated that for those sensitive to DI, amygdala activity scales with DI. We updated the Results (page 19, line 8, page 19, line 16) and the Materials and Methods sections (page 36, line 18) accordingly.

**Comment:** 7.	The vlPFC region highlighted in Figure 3 looks like it might mostly be in white matter - can the authors comment on this? This isn't the region I would normally consider "vlPFC" in a regulatory sense, so I'm not sure how to think about what this region is.

***Response:***

We carefully inspected the peak MNI coordinate in the MRIcroG software and confirmed that the peak lies within the gray matter. We also identified the peak as falling in Brodmann area 47, a canonical subregion of the vlPFC. To show this, we added **S3 Figure**, including the statistical map and an overlaid gray‐matter mask, and Brodmann’s atlas. This figure makes the anatomical context of the peak voxel more transparent. We now think that the reported activity reflects a gray‐matter locus within the previously-established vlPFC and accordingly updated the Results section (page 17, line 20).

**Comment:** 8. The DI regressor in the GLM should be 0 any time there is a fair or advantageous offer. This raises the concern for me that any evidence for differences as a function of DI (especially for PPIs) is largely just tracking whether the offer is 'easy' to accept in the sense of being fair, and thus likely to have a fast RT. If the authors ran a GLM in which there were two onset regressors, one for fair/advantageous trials and one for unfair/disadvantageous trials, with the latter parametrically modulated by the degree of DI, do the results look similar? This would be a more convincing test for the idea that it is sensitivity to the DEGREE of DI that is driving results, rather than just the presence of inequity per se.

***Response:***

We thank the reviewer for pointing out this important potential confounding factor we did not notice previously. In accordance with the suggestion, we separated DI and fair/advantageous conditions and reanalyzed the data using the new GLM. We then applied a parametric modulator of DI magnitude to the former. Response time was also included as a covariate in this individual-level design. We found that the DI‐parametric effect in the dACC remained robustly significant (**Figure 4A**; cluster-level FWE corrected p < 0.05). This indicates that dACC activity scales with the level of DI rather than merely reflecting an “easy vs. hard” division. We explain this change in the Materials and Methods section (page 34, line 10).

**Response to Reviewer #3**

**Comment:** This study investigates the neural correlates of accepting unfair offers during the ultimatum game, thereby complementing many studies that focused more on the neural mechanisms involved in rejection of unfair offers. The authors describe a (standard) fMRI study of the ultimatum game and model rejection behavior with a drift-diffusion model (DDM) that incorporates the level of self-reward and inequality as modulators of the drift rate. The model captures observed behavior, and the level of disadvantageous inequality correlates with ACC activity across trials; moreover, this effect appears stronger in people who accept unfair offers more often. Connectivity analyses furthermore show that functional interactions between ACC and vlPFC were more negative when DI was large, and that vlPFC-amygdala connectivity correlated negatively with rejection rates and response times for acceptance decisions, suggesting that a functional ACC-vlPFC-amygdala circuit is involved in the suppression of internal states related to accepting unfair offers.

The study is interesting in that it examines the acceptance of unfair offers, a behavior that has been much less studied than the rejection of such offers. In general, the study design and methods are solid, and the results should be of interest to the social neuroscience community. However, I have several concerns about the analyses and results that the authors would need to address before their results can be considered conclusive.

***Response:***

We are grateful to the reviewer for the positive evaluation of the original manuscript. As detailed in our responses to the following comments, we have addressed all of the reviewer’s concerns, which greatly helped us make the manuscript more convincing and robust.

**Comment:** (1a)	Initial behavioral analyses: The authors describe that they varied the payoffs from trial to trial by monetary amounts that are not insubstantial (especially compared to the smaller payoffs). However, the initial analyses confirming the behavioral effects of inequality are rather crude: They comprise overall ANOVAs followed by t-tests between the most extreme levels of inequality. This is not convincing; moreover, comparisons of cherry-picked trials (only the most extreme levels) are very prone to selection biases. Please include detailed analyses of how inequality levels affect behavior on all trials, for example regression analyses incorporating the actual payoffs/inequality values. Please do this for both the observed data and the posterior predictive data, to back up your conclusions about the behavioral effects.

***Response:***

As suggested by the reviewer, we examined the contribution of SR, AI, and DI to actual and simulated rejection rates by using a generalized linear mixed model (GLMM). As a result, we found that only DI significantly contributed to both actual (**S1 Table**) and simulated rejection rates (**S2 Table**). We explain these findings in the main text (page 7, line 5 and page 11, line 4) and the Materials and Methods section (page 31, line 6).

**Comment:** (1b)	Also, please report descriptive and statistical analyses of the DDM parameters (e.g., which ones are different from zero?), since these can tell the reader a lot about the behavioral effects of inequality levels on both choices and response times when considered together.

***Response:***

We have added **Table 2**, which provides the group‐level means and standard deviations of the DDM parameters. All drift parameters and boundary separations differ significantly from zero. The starting bias was also significantly different from 0.5. These results are described in the main text (page 10, line 21).

**Comment:** (2)	Specificity of the effects: The authors interpret their results as if the neural activity reflected an emotional mechanism that is uniquely expressed for acceptance of unfair offers. However, the neural analyses link brain activity to the strength of disadvantageous inequality across all trials, irrespective of whether participants accepted or rejected. Please compute GLMs that include choice as a variable and show that the ACC activation is specific for the acceptance of unfair offers, not just correlated with the level of disadvantageous inequality irrespective of choice. Moreover, I wondered to what degree the results generally reflect the level of conflict a participant experiences, rather than specific emotions associated with accepting unfairness? The authors should report whether this is the case, for example by including response time as a covariate in the GLM and examining whether the ACC activation is still present when controlling for general conflict. In any case, this issue should be discussed.

***Response:***

We first separated disadvantageous and advantageous offers. As suggested by the reviewer, we then included response times as a first-level parametric modulator associated with the offer presentation. We found that dACC activity remained stable, while the response time contrast did not produce a significant result (**Figure 2A**). Next, as some participants did not reject any offer, we conducted an additional analysis which included a parametric modulator consisting of choices (to accept or to reject) using the participants who rejected at least once. Even after controlling for the choice, we confirmed similar dACC activity in **S2 Figure** to that in **Figure 4A** under cluster-level family-wised error (FWE) correction (corrected p < 0.05). These results suggest that the dACC activity scales with the magnitude of disadvantageous inequity and is not attributed to general decision conflict, decision time, or choice. We explain these results in the main text (page 14, line 18). Please note that although DI-correlated activity in the dACC and its functional connectivity with the vlPFC are important for accepting unfair offers, we are not claiming this dACC activity is uniquely expressed when accepting unfair offers.

**Comment:** (3)	Selective analysis of only parts of the behavioral data: In the analyses presented in **Figure 2**, the authors report results for "the DI condition". Please report the corresponding results also for the other conditions, and whether the results are specific for DI trials.

***Response:***

In our revised manuscript, we examined whether the relationship between β(DI) and behavior is specific to DI trials or generalizes to other conditions by correlating each participant’s β(DI) with the mean rejection rate and mean response time in AI trials. We found that β(DI) significantly correlated with rejection rates (Pearson’s r = 0.333, Holm-Bonferroni corrected p = 0.0233), while mean response time has no significant correlation with β(DI). These results imply that while the propensity to reject inequity generalizes across DI and AI trials for highly inequity-averse individuals, the processes governing decision speed in the AI condition may be distinct from those in the DI condition. We updated the Results section accordingly (page 13, line 12).

**Comment:** (4)	Selective analysis of only parts of the neural data: Likewise, the analyses presented in Figure 3B are very cherry-picked: They reflect data from a subset of participant selected to show specific effects, and are re-analyses of hand-selected parts of the hemodynamic response for effects used to initially define the ROI. This is bad research practice (double-dipping and cherry-picking). Please report the corresponding analyses as covariations across all participants (with scatterplots), and use temporally unbiased methods to analyze the hemodynamic response.

***Response:***

In accordance with the reviewer’s comment, we divided all participants into high and low β(DI) groups based on the median of their β(DI) parameter estimates. We conducted a two‐way repeated‐measures ANOVA on the PSTH values with factors of Group (high vs. low β(DI)) and Time (each post‐stimulus TR). This analysis revealed significant main effects of Group and Time (**Table 5**), showing that the low β(DI) group consistently exhibited larger ACC responses compared to the high β(DI) group. We also confirmed that the high β(DI) group showed larger PSTH values at 8 seconds (Holm-Bonferroni corrected **p* = 0.0440). We updated **Figure 4B**, the Results (page 15, line 9), and the Materials and Methods sections (page 37, line 14) accordingly.

**Comment:** (5)	Separation of SR, DI, and AI: The GLM contains SR, DI, and AI as parametric modulators. Are they correlated? Can they really be dissociated? The model suggests that, but please report their actual correlations, and the methods you used to make sure the GLM captures only the unique variance (regressor othogonalization).

***Response:***

A standard index to evaluate the co-linearity of variables is the variance inflation factor (VIF; Mumford, 2015). In general, VIF < 10 is regarded as a threshold of independence. We calculated VIF for SR, DI, and AI (**S11 Table**), and all values were found to be much below 5, indicating that collinearity did not distort our parameter estimates. These details were added to the Materials and Methods section (page 29, line 5), with full VIF values in **S11 Table**.

**Comment:** (6)	Behavioral comparison between logistic regression and DDM: It is not surprising that only the DDM but not the regression can capture reaction times, since response times are only used to fit the DDM and do not enter the regression model. This is a feature of the models, not a "better or worse performance". Please discuss this appropriately in the manuscript. Also, please use formal model comparison to see whether for the choice data (acceptance rates) alone, the DDM does better than the logistic regression. Otherwise it is much less justified to say that the logistic performs poorly at capturing the behavior - it may do well at what it is set up to do (explaining choices), but may just not capture response times since these are not included in the model. In any case, the results description and discussion should reflect this qualitative difference between the models.

***Response:***

We thank the reviewer for the suggestions. We did not intend to claim that “DDM outperforms the logistic regression”. We wish here to convey that response times incorporated in the DDM play a crucial role in revealing the involvement of the dACC for accepting unfair offers. Therefore, we removed phrases indicating the categorical superiority of the DDM over the logistic model. In accordance with the reviewer’s suggestion, we compared the DDM and the logistic regression only in terms of choice. We found that logistic regression better explained choices than DDM, revealing the complementary advantages of DDM and logistic regression. We discuss these findings in the Discussion section (page 23, line 7 and line 18).

**Comment:** (7)	Neural comparison between logistic regression and DDM: The authors claim that "a conventional value-based system (i.e., logistic regression) that only considers choices performed poorly compared with the DDM in detecting these fMRI signals". They base this on higher p-values for the same contrast, but never directly compare whether the DDM does better. Please include such a comparison, for example by Bayesian fMRI model comparison or via a GLM that adds both sets of regressors (DDM and logistic) and examines whether the DDM captures unique variance.

***Response:***

In response to the comment, we constructed a second‐level contrast that combined beta values from DDM and logistic regression (see Materials and Methods). This contrast revealed a cluster in the dACC with an uncorrected threshold of 𝑝 < 0.05 (**S6 Figure**). This observation suggests that the DDM model captures additional variance in the dACC responses than the logistic model. We have added this result to the Results section (page 22, line 8) and the Materials and Methods section (page 35, line 5) and include **S6 Figure** to illustrate the contrast map. However, our intention is not to claim the categorical superiority of DDM over value-based models in all contexts. Rather, it is to demonstrate that modeling response time dynamics is essential for revealing any specific ACC engagement observed in our task.

**Comment:** (8)	There are many grammatical errors and typos in the manuscript, please proof read it again.

***Response:***

We apologize for the grammatical errors and typos in the original submission. For the revised manuscript, after a through language check by the authors, we commissioned a professional English editor to proofread the final version.
