# Supplementary material for: The human dorsal anterior cingulate facilitates acceptance of unfair offers and regulates inequity aversion
Source: PLoS Biol. 2026 Feb 5;24(2):e3003007. doi: 10.1371/journal.pbio.3003007 (PMC12875481; doi:10.1371/journal.pbio.3003007)
Supplement: Attachment — Submitted filename: PLOSBioResponseToReviewer_auresp_3.docx [file pbio.3003007.s034.docx]

**Response to Reviewer #2**

**Comment:** I appreciate the work the authors have done to address my previous comments, and I think the paper is much clearer and improved as a result. However, I still had some lingering concerns/suggestions: 1. I appreciated the authors work to address the request for parameter recovery, but I'm afraid that I don't think they've quite satisfied it. Typically, a parameter recovery analysis would examine many iterations of variation across multiple parameters, not just two, and report not only on whether the model seems to get in the ballpark for a given parameter, but also actual correlations and plots of actual vs. estimated parameters, where the ideal is dots falling on a 45 degree line. The reason this is important is because such examination can indicate places and/or parameter values for which the model breaks down, and it can also, crucially, indicate places where parameters 'trade off' against each other (e.g., where a difference in the weight given to, say, DI, is actually picked up in the model by a differences in the starting bias). I don't think the authors need to put this analysis in the main body of the paper, as I think it would be distracting, and their current approach suggests that they would likely find adequate recovery. Perhaps this view is not shared by others, but I would be much more comfortable with the model ability to fit behaviour than is currently the case seeing just two different simulated individuals fit multiple times.

***Response:***

We are grateful to the reviewer for the positive evaluation of our paper and for the insightful feedback. We conducted additional parameter recovery tests by varying our drift parameters $\beta\left( \text{SR} \right)$, $\beta\left( \text{DI} \right)$ and $\beta\left( \text{AI} \right)$, while keeping the boundary, bias, non-decision time at their typical values. As shown in S2 Figure, we found that the estimated $\beta\left( SR \right),$ $\beta\left( \text{DI} \right)$ and $\beta\left( \text{AI} \right)$ plateaued when their true values exceeded 3.0. This does not present a practical problem, as the maximum $\beta\left( \text{SR} \right),$ $\beta\left( \text{DI} \right)$ and $\beta\left( \text{AI} \right)$ across participants were all well below 3.0. We have described these results in the legend of S2 Figure.

**Comment:** 2. I am still uncomfortable with the causal language the authors use, e.g., "Participants who, IN ORDER TO ACCEPT unfair offers suppressed disadvantageous inequity (DI-driven rejection". I think what the authors show here is interesting, but I am still not convinced they can really make the claim that somehow the ACC is SUPPRESSING DI-driven rejection. It is one possible interpretation, and it might be the right one, but there's no causality here. The authors show a correlation, such that ACC representations of DI are stronger when DI is represented LESS in behaviour. They also show some suggestive connectivity analyses (more on this below). But they don't, for instance, MANIPULATE ACC activation through stimulation and show that rejections of inequality go down (or up). Especially since the authors proposed causal ordering (ACC --> vlPFC --> amygdala) implies that amygdala sensitivity to DI should MEDIATE ACC relationships to DI, and the authors don't show this. I think my
proposed solution is for the authors to simply soften their language to admit uncertainty, e.g., "Participants who had lower levels of behavioural disadvantageous inequality aversion actually showed stronger representations of that inequality in the ACC, suggesting that it may play a role in regulating subjective weights." It's a subtle difference, and I'm sorry to keep coming back to this. But the paper is making claims that are just not supportable. What you can show is interesting and suggestive. I'd prefer to just see it described as such, and acknowledged that future work will have to establish causality (which the authors already do).

***Response:*** We agree with the reviewer that our results demonstrate correlations between dACC activity and behavior (as well as the model parameter), rather than causality between them. Following the reviewer’s suggestion, we have revised the abstract accordingly.  The revised sentences now read as follows:

Participants with lower levels of behavioral disadvantageous inequity (DI) aversion (reflected by a lower DDM weight for DI) showed stronger neural representations of DI in the dorsal anterior cingulate cortex (dACC) (page 1, line 23).

These findings suggest that the dACC plays a key role in regulating responses to DI, thereby facilitating the acceptance of unfair offers. (page 1, line 31).

**Comment:** 3. I'd still love to see the authors visualize the negative correlation between ACC representations of DI and behavioural sensitivity to DI differently: can they simply show a scatterplot/correlation plot, where the X-axis is behavioral sensitivity B(DI) and the Y-axis is the estimated beta-coefficient for the ACC response to DI? This would really help to fully interpret ACC responses.

***Response:*** We included the visualization of the negative correlation between ACC representations of DI and behavioral sensitivity to DI in Figure 4C as suggested. We also explained the results in the main text: We confirmed that the beta value for the dACC associated with DI was negatively correlated with β(DI) (**Fig 4C**; Pearson’s $r=-0.549$, Holm-Bonferroni corrected *p* =3.21×10^-6^) (page 13, line 8).

**Comment:** 4. I'm not wholly sure what to make of the amygdala findings as currently reported. It is interesting that amygdala correlates with parametrically with DI in the half of participants who have the strongest B(DI), but that isn't the same thing as saying that, in the full sample, amygdala representations of DI correlate with B(DI) significantly, or that a between-groups t-test of amygdala DI representations are actually different between the high-DI and low-DI group. Maybe this is an issue of noise, since amygdala signals can be harder to measure cleanly perhaps, but I still have a hard time interpreting the relative lack of correlation with individual differences in either vlPFC-amygdala connectivity, or amygdala responsivity, in the story where the amygdala is supposedly the downstream end of a path from ACC-->vlPFC-->amgydala. Can the authors comment on what they think is going on here? Could it actually be the reverse directionality?

***Response:*** Amygdala activity correlated parametrically with DI only in participants who exhibited the strongest behavioral DI aversion (i.e., those with the highest β(DI)), but not in those with weaker aversion. This pattern is consistent with a previous fMRI study showing that prosocial individuals—who strongly dislike inequity and prioritize joint outcomes—displayed a relationship between amygdala activity and inequity, whereas more individualistic individuals did not [7]. Together, these findings suggest a potentially non-linear relationship between inequity aversion and amygdala responses, in which regulatory mechanisms involving the dACC and vlPFC may also contribute. However, further investigation is required to confirm this interpretation, as we did not directly assess social value orientation [7] in the present study. We added this explanation to the discussion section of the revised manuscript (page 22, line 1).

**Comment:** 5. Can the authors clarify whether parametric modulators were or were not orthogonalized with respect to each other, and if so, whether results change if SR is the first variable included in the GLM. This isn't critical, I don't think, but will help to interpret neural parametric correlations (or lack thereof).

***Response:*** We clarified that parametric modulators were not orthogonalized with respect to each other (page 32, line 15).

**Comment:** Smaller comments 6. "The amygdala was reported to respond to inequity in people who dislike it, Line 9 on pg. 19 - can you provide a citation for this?

***Response:*** As suggested, we have added reference [7] in the revised manuscript (page 17, line 3).

**Comment:** 7. For Table 2, can you remind the reader that the upper boundary of the DDM represents rejection. It's in your methods section, but it will help to remind people here to interpret the values of the weights on different quantities.

***Response:*** we have added “The upper boundary of our DDM represents rejection” to the legend of Table 2 (page 44).
